# Mulberry Component Kuwanon C Exerts Potent Therapeutic Efficacy In Vitro against COVID-19 by Blocking the SARS-CoV-2 Spike S1 RBD:ACE2 Receptor Interaction

**DOI:** 10.3390/ijms232012516

**Published:** 2022-10-19

**Authors:** Young Soo Kim, Eun-Bin Kwon, Buyun Kim, Hwan-Suck Chung, Garam Choi, Yeoun-Hee Kim, Jang-Gi Choi

**Affiliations:** 1 Korea Institute of Oriental Medicine, Korean Medicine Application Center, 70 Cheomdan-ro, Dong-gu, Daegu 41062, Korea; 2 R&D Center, Etnova Therapeutics Corp., 198 Saneop-ro, Gwonseon-gu, Suwon 13207, Korea

**Keywords:** coronavirus disease 2019, severe acute respiratory syndrome coronavirus 2, *Morus alba* L., kuwanon C, spike protein, ACE2 receptor

## Abstract

There has been an immense effort by global pharmaceutical companies to develop anti-COVID-19 drugs, including small molecule-based RNA replication inhibitors via drug repositioning and antibody-based spike protein blockers related to cell entry by SARS-CoV-2. However, several limitations to their clinical use have emerged in addition to a lack of progress in the development of small molecule-based cell entry inhibitors from natural products. In this study, we tested the effectiveness of kuwanon C (KC), which has mainly been researched using in silico docking simulation and can serve as an effective building block for developing anti-COVID-19 drugs, in blocking the spike S1 RBD:ACE2 receptor interaction. KC is a natural product derived from *Morus alba* L., commonly known as mulberry, which has known antiviral efficacy. Molecular interaction studies using competitive ELISA and the BLItz system revealed that KC targets both the spike S1 RBD and the ACE2 receptor, successfully disrupting their interaction, as supported by the in silico docking simulation. Furthermore, we established a mechanism of action by observing how KC prevents the infection of SARS-CoV-2 spike pseudotyped virus in ACE2/TPRSS2-overexpressing HEK293T cells. Finally, we demonstrated that KC inhibits clinical isolates of SARS-CoV-2 in Vero cells. Future combinations of small molecule-based cell entry inhibitors, such as KC, with the currently prescribed RNA replication inhibitors are anticipated to significantly enhance the efficacy of COVID-19 therapies.

## 1. Introduction

The coronavirus disease 2019 (COVID-19) pandemic has persisted for 2.5 years despite the development of vaccines and therapeutics for the severe acute respiratory syndrome coronavirus 2 (SARS-CoV-2) pathogen. This is attributable to the emergence of persistent SARS-CoV-2 variants, and is in contrast to the 2009 A/H1N1 pandemic, which was declared post-pandemic by the World Health Organization approximately 1.5 years after the 2009 swine flu outbreak. While the 2009 H1N1 pandemic was quickly contained owing to the early development of the effective, orally administered, small molecule-based drug oseltamivir, better known by its brand name Tamiflu^®^ [1,2], COVID-19 is currently underway since there are no drugs that are as successful in treating this disease as oseltamivir. Inhaled SARS-CoV-2 enters the cell by the binding of its spike S1 receptor-binding domain (RBD) to the angiotensin-converting enzyme 2 (ACE2) receptor on the epithelial cell surface and priming of the spike S2 domain by TMPRSS2, followed by the production of viral genetic material and structural proteins for the release of the assembled SARS-CoV-2 from the cell [3]. Nevertheless, several issues have limited their prescription and use, including the emergence of SARS-CoV-2 resistance to the RNA replication inhibitors remdesivir and paxlovid [4,5,6], unfavorable long-term intravenous administration of antibody-based drugs used to suppress cell entry and remdesivir, paxlovid drug–drug interactions with other medications [7], reproductive toxicity of molnupiravir [8]. Accordingly, further COVID-19 drugs with a variety of targets should be developed. Furthermore, the therapeutic efficacy against COVID-19, particularly against resistant SARS-CoV-2 strains, can be significantly increased by co-administering the RNA replication inhibitors with the drugs having other mechanisms.

Natural products provide valuable building blocks for new antiviral-agent development or prevention of viruses [9,10]. In addition, Natural products can be a safe and cost-effective strategy to protect against various viral infections [11,12]. *Morus alba* L., commonly known as the mulberry plant, has been used as a food source for jams, vinegars, juices, and wines. It is also a traditional medicine with hypoglycemic, hypolipidemic, anti-inflammatory, antiatherogenic, fever-reducing, blood pressure-lowering, and antioxidant efficacies [13,14,15,16,17,18]. The antiviral efficacy of *M.* *alba* L. against herpes simplex virus-1 (HSV-1), influenza virus, and human norovirus (hCoV-229E) has been reported [19,20,21]. Kuwanon C (KC) is a flavone with two isopentenyl groups at positions 3 and 8, which was isolated from the root barks of *M.* *alba* L. [22]. KC has been reported to have a variety of biological activities, such as antiviral, antibacterial, antifungal, tyrosinase inhibitory, antioxidant, neuroprotective, and anti-inflammatory effects [19,23,24,25,26,27,28]. For COVID-19, KC has only been investigated in an in silico docking study with the ACE2 receptor [29]; no in vitro studies of efficacy for preventing clinically isolated SARS-CoV-2 infection by blocking the spike S1 RBD:ACE2 receptor interaction have been performed.

Therefore, in this study, we explored the antiviral mechanism of KC, which was found to inhibit the cell entry of SARS-CoV-2 by blocking the spike S1 RBD:ACE2 receptor interaction based on molecular binding analyses. We verified the effectiveness of KC in vitro in preventing SARS-CoV-2 spike pseudotyped virus and clinically isolated SARS-CoV-2 infection in biosafety level (BSL)-2 and BSL-3 laboratories, respectively.

## 2. Results

### 2.1. KC Blocks Binding between Spike S1 RBD and ACE2 Receptor

We evaluated the potential of KC to block the spike S1 RBD:ACE2 receptor molecular interaction, which is essential for the entry of SARS-CoV-2 into cells, using a competitive ELISA experiment. The effectiveness of the positive control and KC in preventing the molecular binding between the spike S1 RBD and ACE2 receptor was assessed by chemiluminescence using the horseradish peroxidase (HRP) substrate reaction on a spike S1 RBD-coated plate that had been pre-mixed with Fc-tagged ACE2 receptor and positive control anti-spike S1 RBD antibody or KC.

**Figure 1:**
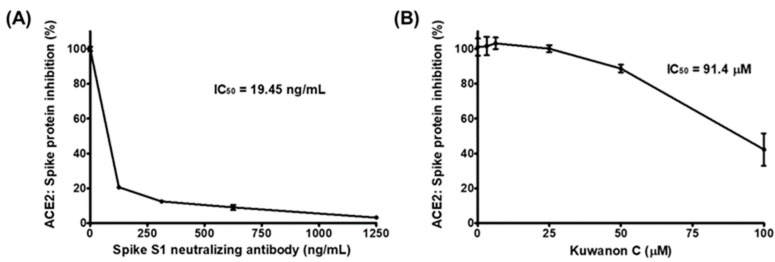
The inhibition of the interaction between the spike protein S1 receptor-binding domain (RBD) and the human angiotensin-converting enzyme 2 (ACE2) receptor by kuwanon C (KC). Spike protein coated on a 96-well plate interacted with a preincubated mixture of the ACE2 receptor and (**A**) anti-SARS-CoV-2 spike S1 antibody as the positive control and (**B**) 0, 3.125, 6.25, 12.5, 25, 50, or 100 μM KC. The inhibition of the spike S1 RBD:ACE2 receptor interaction by KC was determined based on chemiluminescence measurements.

With a 50% inhibitory concentration (IC50) of 19.5 ng/mL, the anti-spike S1 RBD antibody displayed highly effective blocking activity between the coated spike S1 RBD and the treated ACE2 receptor (Figure 1A). KC also demonstrated potential as an inhibitor of SARS-CoV-2 cell entry by gradually decreasing the binding of the ACE2 receptor to the coated spike S1 RBD, with an IC50 of 91.4 µM, in the concentration range from 25 µM to 100 µM (Figure 1B).

### 2.2. Biolayer Interferometry-Based Kinetic Analysis for the Binding of KC to Spike S1 RBD/ACE2 Receptor

**Figure 2:**
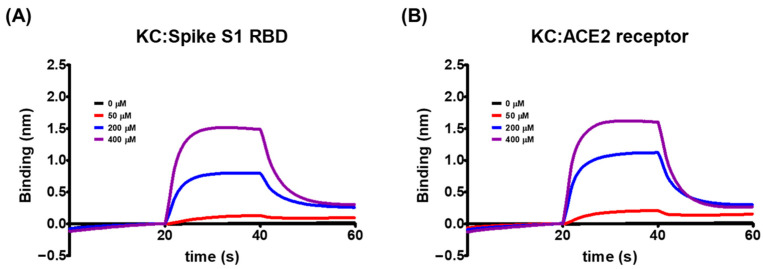
The global kinetic analysis of KC binding to biotinylated (**A**) spike S1 RBD- and (**B**) ACE2 receptor-immobilized BLI sensors. The kinetics for the binding of KC to the spike S1 RBD or the ACE2 receptor were measured by the association of 0, 50, 200, and 400 μM of KC in PBS containing 1% DMSO with immobilized spike S1 or ACE2 receptor and the subsequent dissociation in PBS containing 1% DMSO.

Using the biolayer interferometry-based BLItz system, we explored the molecular binding targets of KC, which was shown in Figure 1 to inhibit the interaction between the spike S1 RBD and the ACE2 receptor. We found that KC binds to both the spike S1 RBD and the ACE2 receptor, when immobilized by biotin–streptavidin binding to the BLI sensor, with equilibrium dissociation constants (K_D_) of 5.03 × 10^−4^ M for the spike S1 RBD and 8.11 × 10^−4^ M for the ACE2 receptor (Figure 2 and Table 1). This minor discrepancy was attributable to KC’s tendency to bind better to and dissociate less slightly from the spike S1 RBD than the ACE2 receptor. As a result of KC’s affinity for both the spike S1 RBD and the ACE2 receptor, KC appears to block the spike S1 RBD:ACE2 receptor interaction by binding to both proteins.

### 2.3. In Silico Docking Simulation and Pharmacophore Analysis of KC with Spike Protein and ACE2 Receptor

We used in silico docking simulation to predict the binding mode and interaction of KC with the spike protein and the ACE2 receptor. The docking of KC (PubChem ID: 5481958) to the spike protein and the ACE2 receptor, respectively, was modeled using AutoDock Vina in the space where both proteins face each other and interact in the spike:ACE2 complex (PDB code: 6M0J). Protein–ligand docking simulation indicated the binding of KC to the spike S1 RBD was more stable than that to the ACE2 receptor, with docking scores (∆G) of −7.1 kcal/mol for the spike S1 RBD and −6.5 kcal/mol for the ACE2 receptor.

**Figure 3:**
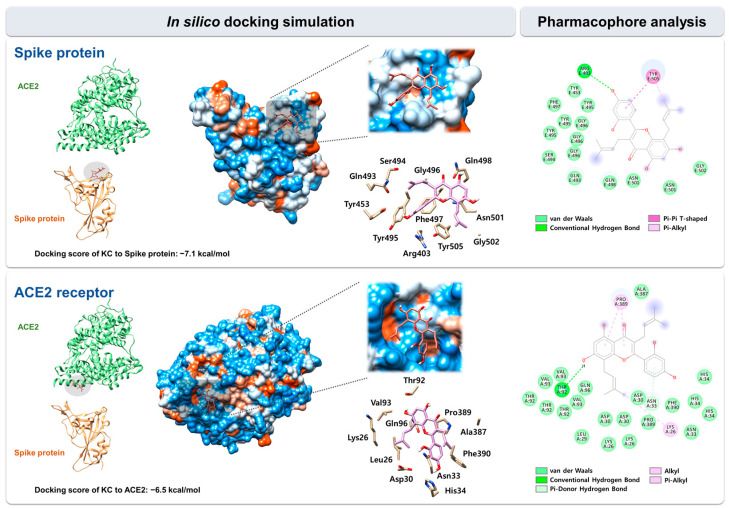
In silico docking simulation between KC and the spike protein/ACE2 receptor. KC was docked onto the SARS-CoV-2 spike protein and ACE2 receptor (PDB code: 6M0J) using AutoDock Vina. The pharmacophore of KC with each target proteins was analyzed using BIOVIA Discovery Studio Visualizer.

Using BIOVIA Discovery Studio Visualizer, pharmacophore analysis on the receptor–ligand binding mode revealed that KC forms 17 and 27 interactions with 11 and 11 amino acids located in the binding interface for the spike S1 RBD and the ACE2 receptor, respectively: spike protein, 14 van der Waals interactions (Tyr453, Gln493, Ser494, Tyr495, Gly496, Phe497, Gln498, Asn501, and Gly502), 1 conventional hydrogen bond (Arg403), 1 pi–alkyl interaction (Tyr505), and 1 pi–pi T-shaped interaction (Tyr505); ACE2 receptor, 22 van der Waals interactions (Lys26, Leu29, Asp30, Asn33, His34, Thr92, Val93, Gln96, Ala387, Pro389, and Phe390), 1 conventional hydrogen bond (Thr92), 1 alkyl interaction (Lys26), 2 pi–alkyl interaction (Pro389), and 1 pi-donor hydrogen bond (Asn33) (Figure 3).

### 2.4. KC Prevents SARS-CoV-2 Lentiviral Pseudovirus from Infecting the ACE2/TMPRSS2-Overexpressing HEK293T Cells

**Figure 4:**
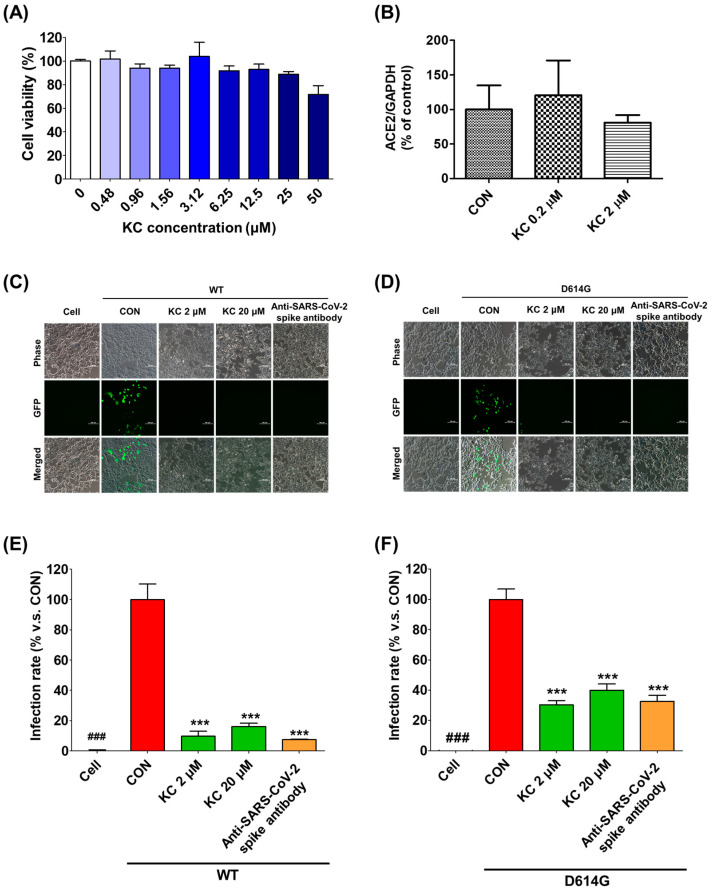
KC inhibits SARS-CoV-2 lentiviral pseudovirus infection in HEK293T cells stably expressing human ACE2 and TMPRSS2. (**A**) The cytotoxic effect of KC in HEK293T cells stably expressing human ACE2 and TMPRSS2 was determined using the MTT assay. HEK293T cells were cultured in 96-well plates (5 × 10^4^ cells/well) for 18 h. (**B**) The ACE2 expression level in HEK293T cells was monitored during KC treatment using real-time quantitative PCR analysis. Then, WT or mutant (D614G) SARS-CoV-2 spike pseudovirus (at a final concentration of 1 × 10^4^ TU/mL to each well) were mixed with different concentrations of KC (2 and 20 μM) or anti-SARS-CoV-2 antibody, and the mixtures were incubated at 37 °C for 1 h. Then, these mixtures were added to HEK293T cells. (**C**,**D**) Green fluorescent protein (GFP) expression levels using flow cytometry were assessed at 72 h after viral infection, scale bar = 100 μm. (**E**,**F**) The inhibitory effect of SARS-CoV-2 spike pseudovirus infection was determined by measuring GFP expression using flow cytometry and measured under a fluorescence microscope. Bar graph (mean  ±  SEM) statistics were determined from three experimental data sets using one-way ANOVA with Tukey’s post hoc test, *** *p*  <  0.001, compared with the CON (KC-untreated) samples. ^###^ *p* < 0.001, compared with the cell-only sample.

We examined the infectivity of pseudotyped SARS-CoV-2, which substitutes the lentiviral VSV-G envelope protein for the SARS-CoV-2 spike protein and contains a gene that can express the GFP protein after infection, when KC was treated to human ACE2/TMPRSSR2-overexpressing HEK293T cells. This experiment aimed to verify the mechanism by which KC inhibited SARS-CoV-2 infection by mediating its binding to the spike S1 RBD and the ACE2 receptor. ACE2/TMPRSS2-overexpressing HEK293T cells were incubated for 24 h with different concentrations of KC (0–50 μM) to determine the noncytotoxic concentration of KC, which was defined as less than 25 μM (Figure 4A). In addition, KC treatment had little effect on the ACE2 expression level in HEK293T cells (Figure 4B).

Subsequently, we assessed the infectivity of pseudotyped SARS-CoV-2 strains with WT and mutated (D614G) spike proteins at concentrations of KC less than 25 μM (2 and 20 μM) (Figure 4C–F). We observed that both pseudotyped SARS-CoV-2 strains penetrate into the ACE2/TMPRSS2-overexpressing HEK293T cells and express green fluorescent protein (GFP) in the KC-untreated group. Similar to the positive control anti-SARS-CoV-2 spike antibody, KC treatment strongly inhibited the spike S1 RBD:ACE2 receptor interaction, resulting in reduced binding between the pseudotyped SARS-CoV-2 and the ACE2 receptor and a significant decrease in GFP expression in the cells. In addition, despite the reports that SARS-CoV-2 with the D614G spike protein mutation had a higher infectivity than that with WT spike protein [30,31,32], KC effectively prevented both pseudotyped SARS-CoV-2 infections at a concentration of 2 μM: 89.9% inhibition in WT and 69.7% inhibition in D614G.

### 2.5. KC Prevents a Clinical Isolate of SARS-CoV-2 from Infecting Vero Cells

**Figure 5:**
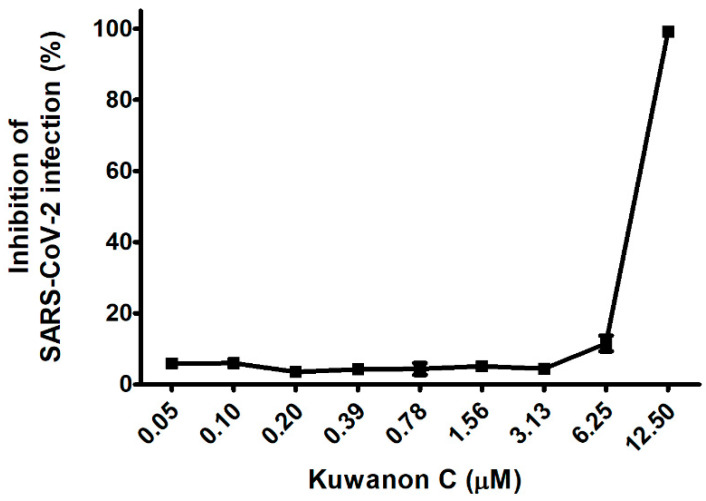
KC suppresses the infection of a clinical isolate of SARS-CoV-2 alpha strain (βCoV/Korea/KCDC03/2020) in Vero cells. Vero cells were cultured on 384-well plates (1.2 × 10^4^ cells/well) for 24 h. Then, Vero cells were infected with SARS-CoV-2 (MOI 0.0125) immediately after being treated with serially diluted KC and incubated at 37 °C for 24 h. The cells were then stained using anti-SARS-CoV-2 nucleocapsid (N) primary antibody, Alexa Fluor 488-conjugated goat antirabbit IgG secondary antibody, and Hoechst 33342.

To further explore our earlier findings, which established the mechanism of action (MoA) by which KC inhibited SARS-CoV-2 infection, we investigated the effect of KC on a clinical isolate of SARS-CoV-2 in African green monkey kidney epithelial cells (Vero cells), which express a high level of the ACE2 receptor. In Vero cells, the cytotoxic effects of KC were measured using Hoechst staining in the concentration range from 0.05 to 12.5 μM, and it was found that KC did not affect cell proliferation up to 12.5 μM. Thereby, the antiviral efficacy of KC against the clinical isolate of SARS-CoV-2 was assessed using an immunofluorescence assay with SARS-CoV-2 nucleocapsid protein antibody and Alexa Fluor 488-conjugated IgG. Vero cells were infected with SARS-CoV-2 at a multiplicity of infection (MOI) of 0.0125 with KC treatment of less than 12.5 μM. While KC did not prevent SARS-CoV-2 infection at concentrations below 3.13 μM, it began to suppress SARS-CoV-2 entry into the cells at concentrations over 3.13 μM. Finally, 99.1% of infection inhibition was demonstrated at a KC concentration of 12.5 μM, and the IC_50_ value of KC for SARS-CoV-2 infection was 7.7 μM (Figure 5).

## 3. Discussion

Despite the widespread vaccination and the development of anti-COVID-19 drugs by global pharmaceutical companies, the COVID-19 pandemic has been ongoing since late 2019 owing to the continued emergence of highly contagious SARS-CoV-2 strains such as Omicron and its sub-variants, resulting in approximately 520 million confirmed cases and 6 million deaths globally. When SARS-CoV-2 is inhaled into the human respiratory tract, the viral spike protein attaches to the ACE2 receptor on the surface of epithelial cells, and the replicated SARS-CoV-2 is subsequently released from the cells. To date, several COVID-19 drugs, including antibody-based spike protein inhibitors and small molecule-based RNA replication inhibitors, have been developed based on the life cycle of SARS-CoV-2. However, the restrictions on their prescription and use in some cases necessitate the continuous development of drugs with higher efficacy and safety: intravenous administration (antibody-based inhibitors and remdesivir); caution in prescribing due to reproductive toxicity (molnupiravir); and drug–drug interactions (paxlovid).

**Figure 6:**
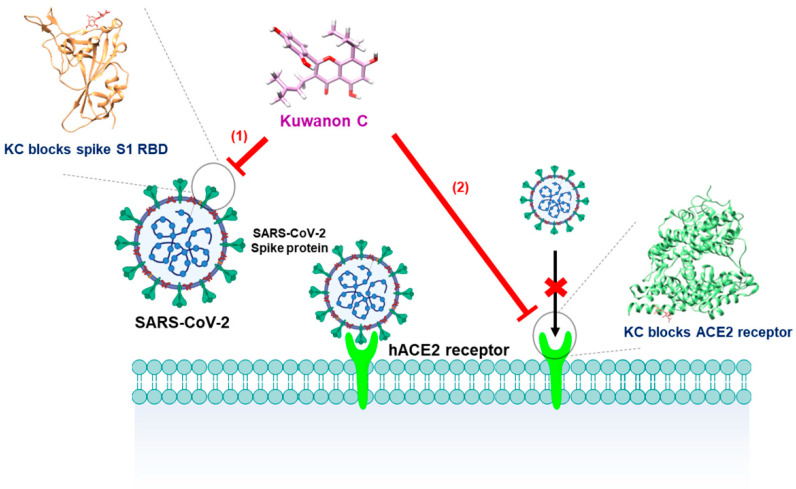
Schematic of the blockade of the SARS-CoV-2 spike S1 RBD:ACE2 receptor interaction by KC.

Natural products contain essential building blocks and active ingredients for developing novel therapeutic agents: the precursor of Tamiflu^®^, shikimic acid from star anise (*Illicium verum*) [33,34], the malaria drug artemisinin from sweet wormwood (*Artemisia annua*) [35,36], and the anticancer drug paclitaxel from yew tree (*Taxus brevifolia*) [37,38]. In particular, *M. alba* L., known as mulberry, may also provide highly efficacious and safe therapeutic candidates because its fruit, twig, leaf, and rood-peel have been used as food and traditional medicines with hypoglycemic, hypolipidemic, anti-inflammatory, antiatherogenic, fever-reducing, blood pressure-lowering, and antioxidant efficacies [13,14,15,16,17,18]. Kuwanon C is a component of *M. alba* L. that has been studied previously for its antiviral, antibacterial, antifungal, tyrosinase inhibitory, antioxidant, neuroprotective, and anti-inflammatory effects [19,23,24,25,26,27,28]; however, no efficacy against COVID-19 has yet been found. In this work, we therefore examined the antiviral activity of KC, which inhibits the spike S2 RBD:ACE2 receptor interaction at the earliest stage of SARS-CoV-2 infection by using molecular binding and in vitro assays (Figure 6).

The binding of the spike S1 RBD to the ACE2 receptor is a crucial step in the entry of SARS-CoV-2 into cells. Using competitive ELISA, we examined the inhibitory efficacy of the molecular binding between the spike S1 RBD and the ACE2 receptor in the presence of KC. A neutralizing antibody against spike S1 RBD was used as the positive control. Reduced chemiluminescence was observed by an increase in the positive control spike S1 neutralizing antibody, showing that the spike S1 neutralizing antibody very efficiently blocked the binding of the ACE2 receptor to the spike S1 RBD coated on a plate (Figure 1A). KC treatment also blocked the spike S1 RBD:ACE2 receptor interaction in a dose-dependent manner, with an IC_50_ of 91.4 µM, at doses up to 100 µM (Figure 1B). We then investigated the binding affinity of KC for the spike S1 RBD and the ACE2 receptor using the BLItz system, and found that KC had equilibrium dissociation constants (*K*_D_) of 5.03 × 10^−4^ M for the spike S1 RBD and 8.11 × 10^−4^ M for the ACE2 receptor (Figure 2 and Table 1). Collectively, most of the natural product-derived inhibitors targeting the spike S1 RBD:ACE2 receptor interaction have been investigated based on in silico simulation [29,39,40,41,42,43]; however, this study not only provides information on a building block of COVID-19 therapeutic agents with the MoA that inhibits SARS-CoV-2 cell entry based on the actual molecular interaction between KC and the spike S1 RBD/ACE2 receptor, it also suggests that further structural analysis of KC derivatives is warranted to obtain increased binding affinity for both proteins.

A previous report showed the binding energy of KC for the ACE2 receptor, the main protease of SARS-CoV-2, and falcipan-2; however, comprehensive research on the molecular interactions of KC is required [29]. Thus, we predicted the binding mode of KC to both the spike protein and ACE2 receptor using in silico binding simulation, as well as the molecular interactions between KC and amino acid residues of each protein in that mode using pharmacophore analysis. Pharmacophore analysis indicated that KC forms 17 and 27 interactions with the spike protein and the ACE2 receptor, respectively (Figure 3). Lan et al. used X-ray crystallography to determine the structure of the spike protein/ACE2 acceptor complex and analyzed the amino acid residues that are located in the hot spots involved in the binding between the two proteins [44]. Amino acids of the SARS-CoV spike protein, including Tyr442, Lew472, Asn479, and Thr487, were revealed as essential amino acids for binding to the ACE2 receptor, corresponding to Leu455, Phe486, Gln493, and Asn501 in the SARS-CoV-2 spike protein. In our pharmacophore analysis, Asn501 of the SARS-CoV-2 spike protein participated in the binding to KC via van der Waals interaction. KC was also predicted to bind to the ACE2 receptor via van der Waals interactions with Asp30 and His34, two of the 20 amino acids located mostly in the ACE2 receptor’s N-terminal helix that are in direct contact with both SARS-CoV-2 and the SARS-CoV spike RBD. The previous study found that Asn30 formed a salt bridge with Lys417 outside of the SARS-CoV-2 spike RBD, unlike Val404 of the SARS-CoV spike RBD, suggesting that this unique interaction may be responsible for the higher binding affinity of the SARS-CoV-2 spike protein for the ACE2 receptor than that of the SARS-CoV spike protein. Both the SARS-CoV-2 and SARS-CoV spike RBDs have also been shown to share eight identical amino acids, including Tyr449, Tyr453, Asn487, Tyr489, Gly496, Thr500, Gly502, and Tyr505, as well as five functionally comparable amino acids, including Leu455, Phe456, Phe486, Gln493, and Asn501. According to our research, KC was bound via van der Waals interactions with the ACE2 receptor’s Asp30/His34 and His34, which interact with the SARS-CoV-2 spike protein’s Leu455 and Gln493, respectively, as well as the SARS-CoV-2 spike protein’s Asn501, which interacts with the ACE2 receptor’s Tyr41, Lys353, Gly354, and Asp355, among the 13 shared amino acids. In addition, Lan et al. reported that polar hydroxyl groups found in the multiple tyrosines of the SARS-CoV-2 spike protein (Tyr449, Tyr489, and Tyr505) interact with ACE2 receptor through hydrogen bonds, which facilitates the binding of the spike protein to the ACE2 receptor. Our docking simulation indicated that one of these residues, Tyr505, may contribute to strong binding of KC through pi–pi T-shaped and pi–alkyl interactions. Omicron variant contains Q493R, G496S, Q498R, N501Y, and Y505H mutations in four amino acids predicted to interact with KC based on in silico docking simulation, indicating that these spike protein mutations may affect KC’s ability to suppress SARS-CoV-2 entry. However, since these mutations in omicron variant do not necessarily indicate the decrease of KC’s inhibitory activity for SARS-CoV-2 entry, further mutagenesis study for each amino acid may reveal alterations in amino acid residues that can affect KC’s inhibitory efficacy. Furthermore, the activity of KC to prevent spike:ACE2 binding can be also improved through a follow-up study based on KC derivatives.

Subsequently, we used the BSL-2 test to assess the antiviral effectiveness of KC against SARS-CoV-2 in vitro by infecting ACE2/TMPRSS2-overexpressing HEK293T cells with pseudotyped SARS-CoV-2, which substitutes the lentiviral VSV-G envelope protein for the SARS-CoV-2 spike protein and contains a gene that can express the GFP protein after infection. ACE2/TMPRSS2-overexpressing HEK293T cells did not exhibit any cytotoxicity at KC concentrations less than 25 μM, and KC had little effect on the ACE2 expression level in HEK293T cells (Figure 4A,B). KC (2 and 20 μM) successfully prevented the entry of pseudotyped SARS-CoV-2 into the cells, which was confirmed by a decrease in GFP fluorescence (Figure 4C–F). This indicated that KC effectively decreases the opportunities for pseudotyped SARS-CoV-2 to bind ACE2/TMPRSS2-overexpressing HEK293T cells by interfering with spike S1 RBD:ACE2 receptor interaction. As previously reported on the higher infectivity of the D614G variant [30,31,32], the D614G mutation decreased the inhibition rate of pseudotyped SARS-CoV-2 infection by KC treatment by 69.7% when compared to the WT strain (89.9% inhibition). The result implies that the continuous evolution in the spike protein may reduce the KC’s ability to prevent SARS-CoV-2 entry. Nonetheless, KC’s backbone can be used in developing therapeutic agents for COVID-19 because KC still has the potential to prevent SARS-CoV-2 entry. Therefore, further studies using orthogonal biophysical assays and KC derivatives will help characterize the binding of KC to S1 RBD and ACE2 and develop new COVID-19 therapeutics with improved efficacy.

Finally, KC‘s antiviral effectiveness against COVID-19 was verified by examining a clinical isolate of SARS-CoV-2 from infecting Vero cells. At doses that were not cytotoxic to Vero cells, the KC therapy suppressed viral infections in Vero cells almost completely at 12.5 μM with an IC_50_ value of 7.7 μM (Figure 5). Collectively, our result showed that KC prevents the early stage of SARS-CoV-2 infection by targeting both the spike S1 RBD and the ACE2 receptor, unlike the commercially available small molecule-based COVID-19 drugs that inhibit RNA replication, suggesting the potential of KC as an inhibitor that hinders the penetration of clinically isolated SARS-CoV-2. Nonetheless, drug–drug interactions can complicate drug development and prescription. The ACE2 receptor is also involved in the renin–angiotensin system (RAS) which plays an important role in regulating blood pressure as well as cancer progression. The ACE inhibitor hypertension drug relieves vasoconstriction by inhibiting ACE expression and increasing ACE2 expression, which restricts the conversion of angiotensin I to angiotensin II [45,46,47]. Furthermore, ACE2 expression is inversely related to tumor angiogenesis and progression in many cancers [48,49,50,51,52]. Therefore, future study on KC in vivo should closely monitor RAS regulation and cancer prognosis, which may be caused by KC binding to the ACE2 receptor. In addition, Kumari et al. suggested that KC may act as an inhibitor for the main protease of SARS-CoV-2 and falcipain-2 involved in SARS-CoV-2 replication as well as ACE2 receptor binding through in silico molecular docking analysis [29], indicating that KC may be effective against COVID-19 by blocking SARS-CoV-2 entry via ACE2 receptor binding and inhibiting SARS-CoV-2 replication. However, since the prediction based on in silico analysis does not necessarily correlate with actual experimental results, it is required to examine the anti-COVID-19 efficacy of KC via other antiviral mechanisms using the suitable cell lines and KC treatment method. Therefore, the integration of this work and further research can suggest a comprehensive anti-COVID-19 mechanism of KC throughout the SARS-CoV-2 life cycle. Furthermore, similar to the cocktail therapy for acquired immune deficiency syndrome, the combination of a commercially available RNA replication inhibitor and a SARS-CoV-2 cell entry inhibitor, such as KC and its derivatives, will be able to increase antiviral effectiveness, not only against the current SARS-CoV-2 strains, but also against mutant and resistant SARS-CoV-2 strains [53,54].

## 4. Materials and Methods

### 4.1. Materials

KC was purchased from ChemFaces (Wuhan, China). The SARS-CoV-2 spike/ACE2 inhibitor screening assay kit, biotin-labeled recombinant protein ACE2 receptor, and spike protein S1 RBD (BPS Bioscience, San Diego, CA, USA) were purchased for the competitive ELISA assay and BLItz analysis. Human ACE2/TMPRSS2-overexpressing HEK293T stable cell line was purchased from GeneCopoeia (Rockville, MD, USA) and maintained in Dulbecco’s modified Eagle’s medium (Lonza, Walkersville, MD, USA) containing 10% fetal bovine serum (Biotechnics Research, Lake Forest, CA, USA) and 1% penicillin/streptomycin (Cellgro, Manassas, VA, USA) at 37 °C in a 5% CO_2_ incubator. WT and mutant (D614G) SARS-CoV-2 spike pseudotyped viruses (cat. no. SP101-100 and SP103-100) were purchased from GeneCopoeia (Rockville, MD, USA). Vero cells were purchased from American Type Culture Collection (Manassas, VA, USA). A clinical isolate of SARS-CoV-2 alpha strain (βCoV/Korea/KCDC03/2020) was provided by Korea Centers for Disease Control and Prevention (Cheongju, Korea).

### 4.2. SARS-CoV-2 Spike/ACE2 Inhibitor Screening Assay

The binding of KC to the SARS-CoV-2 spike protein and the ACE2 receptor was analyzed using the SARS-CoV-2 spike/ACE2 inhibitor screening assay kit (BPS Bioscience, San Diego, CA, USA). Each well of a 96-well plate was coated with 50 μL of 1 μg/mL SARS-CoV-2 spike protein in PBS overnight at 4 °C and then washed with 100 μL of 1× immune buffer. After the addition of 100 μL of 1× blocking buffer and incubation at room temperature for 1 h, the plate was washed again. Subsequently, each well was treated with 20 μL of 1× immune buffer, followed by 10 μL of inhibitor solution containing the SARS-CoV-2 spike protein antibody (Active Motif, Carlsbad, CA, USA) or KC. After incubation with slow shaking at room temperature for 1 h, a mixture of 20 μL of 2.5 μg/mL ACE2 inhibitor solution containing KC or SARS-CoV-2 spike antibody was added and incubated for 1 h at room temperature with slow shaking, then washed three times with 100 μL of 1× immune buffer. Subsequently, the plate was applied to each well with 100 μL of 1× blocking buffer, incubated at room temperature for 10 min, and then washed again. After, each well was added with 100 μL of anti-His-HRP and incubated for 1 h. The plates were washed three times with 1× immune buffer, blocked with 100 μL of 1× blocking buffer per well at room temperature for 10 min, and washed again. Relative chemiluminescence was measured using a GloMax-multi microplate reader (Promega, Madison, WI, USA).

### 4.3. Kinetic Analysis of the Binding between KC and Spike Protein/ACE2 Receptor Based on Biolayer Interferometry

Using a BLItz system (Sartorius AG, Göttingen, Germany), the binding affinities and kinetics of KC for the spike protein RBD and the ACE2 receptor were assessed. The streptavidin BLI sensors (Sartorius AG, Göttingen, Germany) were pre-equilibrated in PBS buffer for 10 min; then, biotinylated spike protein S1 RBD and ACE2 receptor were immobilized on the BLI sensors by soaking the sensor in 4 μL of 50 μg/mL spike protein RBD and ACE2 receptor solution. The KC solutions (at 0, 50, 100, 200, and 400 μM) were prepared by dissolving KC in PBS containing 1% DMSO.

At first, the BLI sensor immobilized with each protein was equilibrated in PBS buffer containing 1% DMSO for 10 s. Then, 4 μL of each concentration of KC solution was associated with the equilibrated protein-immobilized sensor for 10 s, followed by dissociation in PBS containing 1% DMSO for 10 s. The kinetic constants were calculated using BLItz Pro by fitting the association and dissociation data to a 1:1 model. The equilibrium dissociation constant, *K*_D_, was calculated as the dissociation constant divided by the association constant (*k*_d_/*k*_a_).

### 4.4. In Silico Docking Simulation and Pharmacophore Analysis

KC (PubChem ID: 5481958) was used as a ligand. KC was docked in a space where the SARS-CoV-2 spike protein and the ACE2 receptor face each other (PDB code: 6M0J). The binding modes and binding affinities of KC to the spike protein and the ACE2 receptor were obtained using AutoDock Vina integrated with UCSF Chimera 1.15 (accessed on 15 June 2022; https://www.cgl.ucsf.edu/chimera/). The binding affinity was presented as the lowest energy score in the docking simulation. The pharmacophore analysis between KC and the spike protein/ACE2 receptor was performed and visualized as receptor–ligand interactions on a 2D diagram in BIOVIA Discovery Studio Visualizer.

### 4.5. Cell Viability Assay

Cell viability was determined using the 3-[4,5-dimethylthiazol-2-yl]-2,5 diphenyl tetrazolium bromide (MTT) assay. ACE2/TMPRSS2-overexpressing HEK293T cells were seeded into 96-well plates at 5 × 10^4^ cells/well, and KC was added to each well at concentrations of 0–50 μM. MTT solution was applied to each well after 24 h, and the cells were incubated for 30 min. Then, 200 μL of dimethyl sulfoxide was added per well, and the absorbance at 540 nm was measured using an Epoch Microplate Reader (BioTek, Winooski, VT, USA). The data were presented as the mean ± SEM of four independent experiments.

### 4.6. SARS-CoV-2 Lentiviral Pseudovirus Infection Assay

The SARS-CoV-2 spike pseudotyped lentivirus infection assay was performed in ACE2/TMPRSS2-overexpressing HEK293T cells. ACE2/TMPRSS2-overexpressing HEK293T cells were cultured in 96-well plates (5 × 10^4^ cells/well) for 18 h. WT and mutant (D614G) SARS-CoV-2 spike pseudovirus (at a final concentration of 1 × 10^4^ TU/mL to each well) was incubated with 2 and 20 μM KC or anti-SARS-CoV-2 spike antibody at 37 °C for 1 h, which were then added to ACE2/TMPRSS2-overexpressing HEK293T cells. The SARS-CoV-2 spike pseudovirus infection was determined at 3 days after infection by measuring GFP expression using flow cytometry and fluorescence microscopy (Olympus, Tokyo, Japan). For flow cytometry, cells were harvested via trypsinization and move to microcentrifuge tubes. The pellet cells at 400× *g* for 5 min and wash third time with 3% BSA in PBS. After the final wash, resuspend in 1% BSA in PBS and analyze via flow cytometry (Olympus, Tokyo, Japan).

### 4.7. RNA Isolation and Real-Time PCR

Total RNA isolation and cDNA synthesis were carried out using the TRIzol™ Reagent (Thermo Scientific, Rockford, IL, USA) and cDNA using the Maxima First Strand cDNA Synthesis Kit for RT-qPCR (Thermo Fisher Scientific, Waltham, MA, USA) according to the supplier’s instructions, respectively. The primer sequences used are ACE2 (F: 5′-TCCATTGGTCTTCTGTCACCCG-3′ and R: 5′-AGACCATCCACCTCCACTTCTC-3′) and β-actin (F: 5′-GGAAATCGTGCGTCACATCA-3′ and R: ATCTCCTGCTCGAAGTCCAG-3′). The PCR cycle was as follows: 95 °C for 10 min, 40 cycles of 95 °C for 20 s, 55 °C (ACE2 and β-actin) for 40 s, and at the end of each experiment, a melting curve analysis was performed to confirm that a single product per primer pair was amplified. Amplification and analysis were performed using the C1000 TouchTM Thermal Cycler (Bio-Rad, Hercules, CA, USA), and each sample was compared using the relative CT method. Fold changes in gene expression were determined relative to the blank control after normalization to β-actin expression using the 2−ΔΔCt method.

### 4.8. The Prediction of Physicochemical Properties of KC

The physicochemical properties, including drug-likeness and pharmacokinetics, of KC were predicted using SWISSADME, an online web-server (accessed on 20 September 2022; http://www.swissadme.ch/) [55].

### 4.9. Statistical Analysis

The data are presented as the mean ± standard error of the mean and were analyzed using GraphPad PRISM software (v5.02; GraphPad, San Diego, CA, USA).

## 5. Conclusions

This study demonstrated that KC, a component of *M. alba* L., exerts antiviral activity by preventing SARS-CoV-2 from attaching to cells. This suggests that SARS-CoV-2 infection, when originally introduced into the respiratory system, as well as re-infection by replicated SARS-CoV-2 may be successfully inhibited by KC. The rational design of promising KC derivatives using in silico simulation will significantly improve the antiviral efficacy of small molecules with KC’s backbone. Nonetheless, verification based on molecular and in vitro assay is necessary because in silico analysis does not necessarily reflect actual intermolecular interactions. In addition, further investigation using a human ACE2 transgenic mouse model infected with SARS-CoV-2 in an animal biosafety level 3 laboratory is warranted to verify the antiviral efficacy of KC and its derivatives as cell entry inhibitors in vivo.

## Figures and Tables

**Table 1:** The binding kinetics of KC to spike S1 RBD and ACE2 receptor.

	*K*_D_ (M)	*k*_a_ (M^−1^ s^−1^)	*k*_d_ (s^−1^)	*R* ^2^
Spike S1 RBD	5.03 × 10^−4^	4.24 × 10^2^	2.13 × 10^−1^	0.9924
ACE2 receptor	8.11 × 10^−4^	3.15 × 10^2^	2.56 × 10^−1^	0.9922

## Data Availability

The data that support the findings of this study are available from the corresponding author upon reasonable request.
